# Impact of a psychoeducational nursing program on self-efficacy and psychological adjustment in hemodialysis patients

**DOI:** 10.1038/s41598-025-04052-x

**Published:** 2025-06-04

**Authors:** Basmah Abdel-Nasser Helali, Sahar Mahmoud Eliwa, Fatma Ata Abd El-Salihen Nosir, Shimaa Saied Adam Mohamed

**Affiliations:** Psychiatric and Mental Health Nursing, Faculty of Nursing, Ain Shams University, Cairo, 11566 Egypt

**Keywords:** Psychoeducational, Self-efficacy, Psychological adjustment, Hemodialysis, Psychology, Health care, Medical research, Nephrology

## Abstract

Self-efficacy and psychological adjustment to illness are crucial for new hemodialysis patients as they empower individuals to manage their treatment effectively and cope with the emotional and physical challenges of their condition. Research on the psychological aspects of hemodialysis patients in the Middle East is limited, and even rarer are interventions specifically tailored to promote them. This study evaluates the impact of a psychoeducational nursing program on self-efficacy and psychological adjustment among hemodialysis patients. A quasi-experimental design was utilized with a purposive sample of 53 patients aged 18–60 years, undergoing hemodialysis for less than a year at NIUN, Cairo, Egypt. Data were collected using a socio-demographic and clinical data questionnaire, Self-Efficacy for Hemodialysis Scale, and Psychological Adjustment to Illness Scale. Our program, which specifically targeted new hemodialysis patients, aimed to facilitate faster and more effective psychological adaptation through interactive discussions, practical coping strategies, and educational materials on disease management and self-care. Our study showed improvement in self-efficacy for hemodialysis patients in pre- and post-program implementation, with a p-value of (0.057), and a highly significant improvement in psychological adjustment to illness, with a p-value of (0.001). Based on the improvements observed, psychoeducational programs should be integrated into standard care for new hemodialysis patients, with a focus on multidisciplinary collaboration to address both medical and psychological needs.

## Introduction

New hemodialysis patients often face a steep learning curve as they adjust to their new treatment regimen. The abrupt lifestyle changes, including strict dietary restrictions and fluid management, can be particularly challenging^1^. In addition to physical symptoms like fatigue and discomfort, these patients experience significant emotional distress and anxiety as they grapple with the sudden shift in their health and well-being^2^.

Self-efficacy, or one’s confidence in performing specific behaviors, is notably impaired in patients undergoing hemodialysis, similar to other chronic conditions like diabetes, hypertension, and chronic lung disease. It is a key psychological factor that not only facilitates behavioral change but also significantly enhances treatment adherence and leads to better health outcomes. High self-efficacy has been associated with increased confidence in managing health behaviors, such as adhering to treatment regimens, and plays a crucial role in both initiating and maintaining these behaviors^3^.

For hemodialysis patients, the level of self-efficacy is closely linked to their positive beliefs about their disease, health outcomes, and treatment compliance. Patients with greater self-efficacy tend to report better psychological adjustment, which in turn contributes to their overall well-being. Despite its importance, self-efficacy in this patient group remains underexplored, highlighting the need for research into effective interventions that can enhance self-efficacy and improve patient outcomes in hemodialysis settings^4^.

## Literature review

Emerging evidence suggests that self-efficacy plays a critical role in the adjustment of hemodialysis patients. It is strongly associated with improved autonomy, better symptom management, and enhanced well-being. Specifically, self-efficacy helps patients manage the challenges of hemodialysis more effectively, leading to better adherence to treatment and greater overall functioning^5^.

Moreover, psychological adjustment is crucial for hemodialysis patients, as they often face significant emotional challenges, including anxiety, depression, and stress, due to the disruption of their daily lives. The requirement for regular hemodialysis sessions, combined with lifestyle changes and concerns about long-term health, can lead to emotional instability. Successful adjustment not only enables better condition management but also enhances emotional resilience^6,7^.

Psychoeducational interventions that combine education, emotional support, and practical guidance have proven effective in improving both psychological adjustment and treatment adherence across various chronic conditions, including cancer and stroke. These interventions have been systematically assessed and shown to be beneficial in enhancing patient understanding, engagement, and well-being^8^.

Although psychoeducational interventions have shown promising benefits in managing chronic illnesses, their application to hemodialysis patients remains limited, particularly in the context of enhancing self-efficacy and psychological adjustment. This study seeks to fill this gap by evaluating the effectiveness of a psychoeducational nursing program specifically designed for hemodialysis patients. By focusing on these key psychological outcomes, which are essential for improving treatment adherence and overall well-being, this research offers valuable insights into a critical area of patient care that has been underexplored in the existing literature.

## Results

### Sociodemographic and clinical data of studied hemodialysis patients

Table 1 illustrates the socio-demographic and clinical characteristics of the study participants. The majority (35.8%) of participants were aged between 40 and 50 years, with a mean age of 44.9 years (± 10.68). A higher proportion of participants were male (64.2%) compared to female (35.8%). Regarding marital status, most participants were married (73.6%). In terms of education, 49.1% had completed higher education, while 47.2% had middle education. Regarding the duration of hemodialysis, 39.6% of participants had been on hemodialysis for less than six months, with a mean duration of 6.55 months (± 3.15). Additionally, 49.1% of participants were employed as technicians, 35.8% were professionals, and 5.7% were either students or unemployed.

**Table 1:** Socio-demographic and clinical characteristics of the study participants.

Socio-demographic & clinical data	No	%
Age (years)		
< 40 years	16	30.2
40– < 50 years	19	35.8
50– < 60 years	13	24.5
≥ 60 years	5	9.4
Mean ± SD	44.90 ± 10.68	
Gender		
Male	34	64.2
Female	19	35.8
Marital status		
Single	9	17.0
Married	39	73.6
Divorced	3	5.7
Widowed	2	3.8
Level of education		
Reads & Write	2	3.8
Middle Education	25	47.2
High Education	26	49.1
Date since starting hemodialysis		
< 6 months	21	39.6
6– < 10 months	19	35.8
≥ 10 months	13	24.5
Mean ± SD	6.55 ± 3.15	
Profession		
Student/unemployed	3	5.7
Technician	26	49.1
Professional	19	35.8
Others	5	9.4

### Self-efficacy among studied hemodialysis patients

**Fig. 1:**
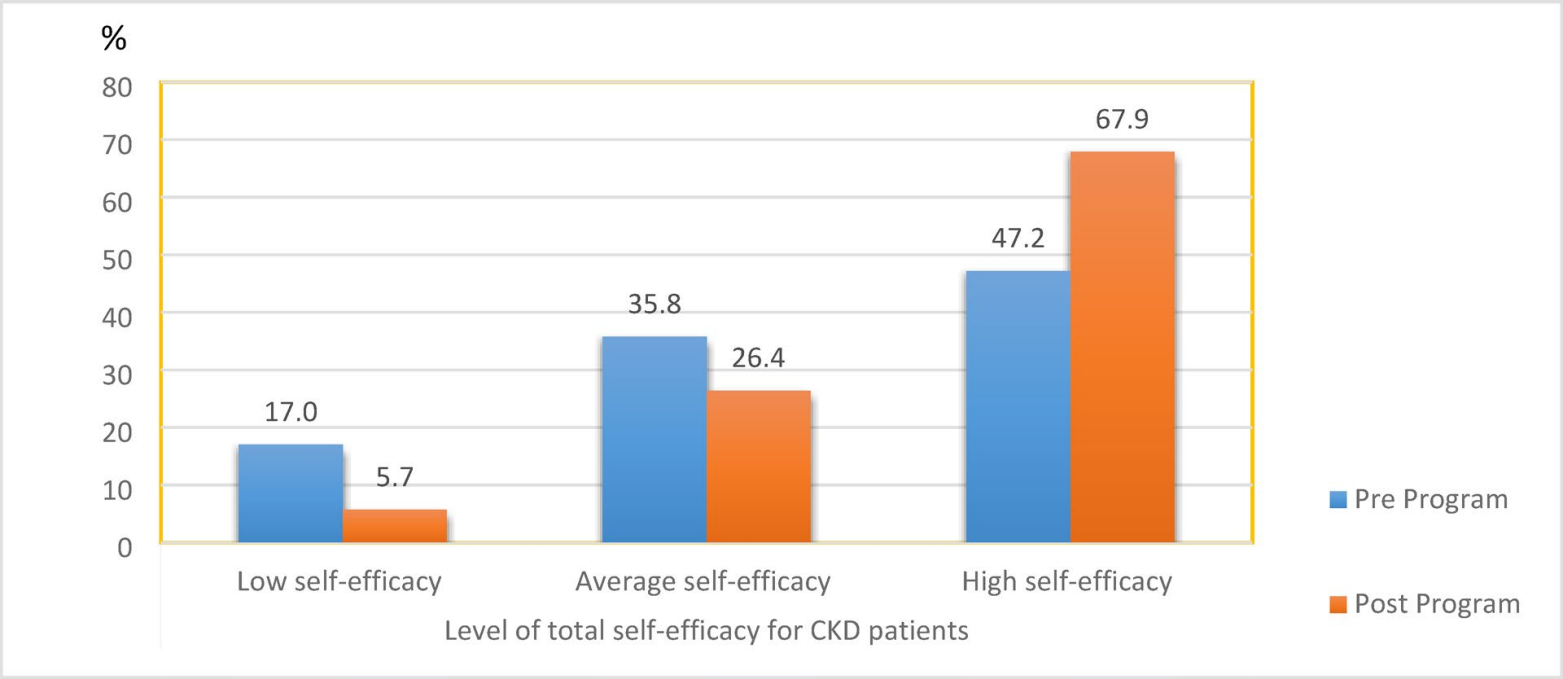
Distribution of the studied patients’ level of self-efficacy pre and post program.

Figure 1 shows the distribution of self-efficacy levels among hemodialysis patients before and after the program implementation. The number of patients with low self-efficacy decreased from 17.0% pre-program to 5.7% post-program, while the proportion with high self-efficacy increased from 47.2 to 67.9%. The chi-square test revealed a trend towards statistical significance (χ^2^ = 5.741, *p* = 0.057) in the change of self-efficacy levels.

Table 2 illustrates the changes in self-efficacy across various domains for hemodialysis patients before and after the program implementation. A significant increase was observed in the problem-solving subdomain, increasing from 41.5 to 66.0% (*p* = 0.009). Similarly, in the autonomy subdomain, the percentage increased from 52.8 to 73.6% (*p* = 0.068). While improvements were also noted in the self-integration, seeking social support, and identifying and managing emotions subdomains, these changes were not statistically significant, with *p* values of 0.099, 0.267, and 0.051, respectively.

**Table 2:** Distribution of self-efficacy domains in hemodialysis patients pre and post program implementation.

Domains	Pre program						Post program						Pre-post	
	Disagree		Sometimes		Agree		Disagree		Sometimes		Agree		*x*^*2*^	*p* value
	No	%	No	%	No	%	No	%	No	%	No	%		
Autonomy	10	18.9	15	28.3	28	52.8	4	7.5	10	18.9	39	73.6	5.377	0.068
Self-integration	6	11.3	23	43.4	24	45.3	2	3.8	17	32.1	34	64.2	4.624	0.099
Problem solving	8	15.1	23	43.4	22	41.5	1	1.9	17	32.1	35	66.0	9.309	0.009
Seeking social support	7	13.2	15	28.3	31	58.5	3	5.7	12	22.6	38	71.7	2.643	0.267
Identifying and managing own emotions	15	28.3	19	35.8	19	35.8	6	11.3	18	34.0	29	54.7	5.968	0.051

### Psychological adjustment to illness among studied patients

**Fig. 2:**
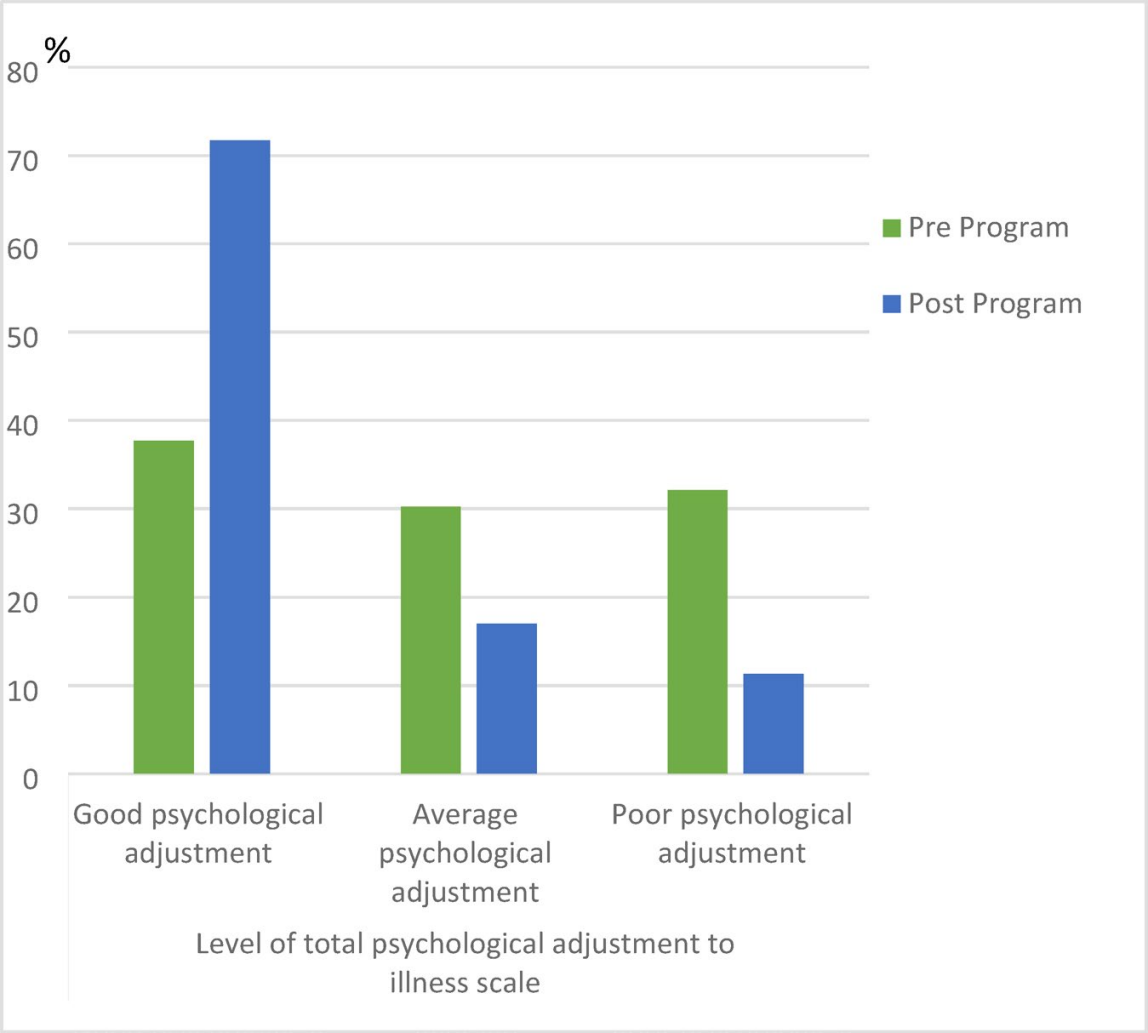
Distribution of the studied patients’ psychological adjustment to illness pre and post program implementation.

Figure 2 illustrates the changes in the psychological adjustment to illness scale before and after the program. The percentage of patients with good psychological adjustment increased significantly from 37.7% pre-program to 71.7% post-program (χ^2^ = 12.807, *p* < 0.001), while those with poor psychological adjustment decreased from 32.1% to 11.3%.These results indicate a significant overall improvement in psychological adjustment following the program.

Table 3 shows the changes in psychological adjustment to illness subdomains before and after the program. The subdomain of healthcare orientation significantly increased from 41.5 to 84.9% (*p* < 0.001), the vocational environment subdomain increased from 43.4 to 69.8% (*p* = 0.017), and domestic environment improved from 45.3 to 81.1% (*p* < 0.001). Similarly, extended family relationships increased from 43.4 to 71.7% (*p* = 0.013), the social environment increased from 28.3 to 58.5% (*p* = 0.007), and psychological distress improved from 34.0 to 62.3% (*p* = 0.009). These results demonstrate significant improvements in all domains following the program.

**Table 3:** Distribution of the studied patients according to domains of psychological adjustment for hemodialysis patients of (pre/ post) program (N = 53).

Domains	Pre program						Post program						Pre-post	
	Good		Average		Poor		Good		Average		Poor		*x*^*2*^	*p* value
	No	%	No	%	No	%	No	%	No	%	No	%		
Healthcare Orientation	22	41.5	15	28.3	16	30.2	45	84.9	7	13.2	1	1.9	24.040	< 0.001**
Vocational Environment	23	43.4	17	32.1	13	24.5	37	69.8	11	20.8	5	9.4	8.108	0.017**
Domestic Environment	24	45.3	18	34.0	11	20.8	43	81.1	7	13.2	3	5.7	14.799	< 0.001**
Extended family relationships	23	43.4	17	32.1	13	24.5	38	71.7	9	17.0	6	11.3	8.729	0.013*
Social Environment	15	28.3	15	28.3	23	43.4	31	58.5	10	18.9	12	22.6	10.022	0.007*
Psychological Distress	18	34.0	12	22.6	23	43.4	33	62.3	10	18.9	10	18.9	9.715	0.009*

### Correlation between self-efficacy and psychological adjustment to illness among hemodialysis patients

Table 4 shows that there is a highly significant correlation between self-efficacy and psychological adjustment to illness among hemodialysis patients in pre and post program.

**Table 4:** Correlation between total score of total score of self-efficacy for hemodialysis patients and total score of psychological adjustment to illness for (pre-post program). (N = 53).

	Total score of self-efficacy for hemodialysis patients (Pre)	Total score of psychological adjustment to illness (Pre)	Total score of self-efficacy for hemodialysis patients (Post)	Total score of psychological adjustment to illness (Post)
Total score of self-efficacy for CKD patients				
r-value		0.485		0.737
*p* value		< 0.001**		< 0.001**
N		53		53
Total score of psychological adjustment to illness scale				
r-value	0.485		0.737	
*p* value	< 0.001**		< 0.001**	
N	53		53	

## Discussion

Enhancing psychological adjustment and self-efficacy is essential for improving the well-being of patients undergoing hemodialysis. Although hemodialysis compensates for lost kidney function but it also presents significant physical, emotional, and social challenges. Patients often struggle with adapting to these demands, which can negatively impact their quality of life. Structured psychoeducational interventions offer a promising approach to strengthening psychological resilience and equipping patients with effective coping strategies. This study examines the impact of a psychoeducational nursing program designed to support hemodialysis patients in managing their condition more effectively.

Regarding sociodemographic and clinical characteristics, the study found that most new hemodialysis patients were male, married, and between 40 and 50 years old. This aligns with higher lifestyle risk factors in men, such as smoking, overuse of analgesics, and a greater prevalence of hypertension and diabetes, which can lead to delayed diagnoses and the need for dialysis. These findings are consistent with those of Farag and ElSayed^9^, who reported that most hemodialysis patients were male and aged 55 years or older.

Since sociodemographic factors can influence psychological adjustment and self-efficacy, understanding these characteristics is essential for interpreting patients’ responses to psychoeducational interventions. Differences in age, gender, and social support may shape how individuals engage with and benefit from the program, ultimately impacting their adaptation to hemodialysis and treatment adherence.

In addition, most patients were employed in technical professions with middle to high levels of education, which may be linked to lifestyle factors and job-related stress. These results mirror findings from Nuñez^10^, who observed similar patterns in employment and education among hemodialysis patients.

Regarding self-efficacy, our study found a significant improvement in the overall level of self-efficacy among new hemodialysis patients. Prior to the program, fewer than half of the patients demonstrated a high level of self-efficacy; however, this number increased to more than two-thirds following program implementation. These results are consistent with those of Lee et al.^11^, who reported that the majority of hemodialysis patients exhibited high self-efficacy and that intervention programs significantly improved self-efficacy among new hemodialysis patients.

In addition, our study revealed a highly significant improvement in the autonomy and self-integration subdomains of self-efficacy before and after the program implementation. Prior to the program, half of the participants exhibited high autonomy, which increased to nearly three-quarters following the program. Similarly, fewer than half of the participants demonstrated high self-integration before the program, with this figure rising to more than two-thirds in the post-program evaluation. But given the already high baseline score in self-integration subdomain, the improvement was not as substantial as other dimensions. These results are consistent with those of Almutary and Tayyib^12^, who found that hemodialysis patients exhibited moderate to high levels of autonomy and self-integration.

These improvements underscore the critical role of psychoeducational interventions in fostering self-efficacy and overall well-being in hemodialysis patients. By equipping patients with essential skills and knowledge, such programs not only support better disease management but also contribute to more substantial personal growth and adaptation.

Moreover, our study found a significant improvement in the problem-solving and identifying and managing emotion subdomains of self-efficacy before and after the program implementation. However, limited improvement was observed in the seeking social support subdomain. Prior to the program, the majority of patients demonstrated a moderate level of problem-solving ability, which increased to a high level after the program.

In contrast, while less than two-thirds of the patients reported a high level of seeking social support before the program, it rose to nearly three-quarters in the post-program evaluation. The lack of significant improvement in the seeking social support subdomain may be attributed to several factors. For instance, patients may have had established social support networks prior to the intervention, limiting the potential for further improvement. Additionally, cultural or personal barriers, such as reluctance to seek support or difficulty in forming new support networks, could have impacted this outcome.

Before the program, approximately one-third of patients had moderate to high emotional management skills, which increased to over half after the program. This significant improvement highlights the importance of incorporating comprehensive emotional and coping skills training into psychoeducational programs. Addressing these areas, equips patients to better manage the emotional complexities of their condition and enhance their overall quality of life. These findings are similar to the findings of Hafezieh et al.^13^, whose findings showed that hemodialysis patients had high level of problem solving and moderate level of emotional management.

About psychological adjustment to illness, our study findings showed a highly significant improvement in total psychological adjustment to illness among hemodialysis patients in pre and post program implementation. In pre-program implementation, only one third of the patients had high level of psychological adjustment to illness that increased to nearly three quarters of the patients in post program evaluation. This indicated the effectiveness of our psychoeducational nursing program that addressed identifying and facing negative thoughts, coping strategies, relaxation techniques, therapeutic communication, and problem-solving, which provided the patients with a holistic toolkit to effectively manage the challenges associated with their illness.

These findings are consistent with the findings of Barello et al.^14^ and Kapadi et al.^15^, whose findings suggested that psychoeducational interventions caused a noticeable improvement in psychological wellbeing/adjustment among hemodialysis patients and that hemodialysis patients had successful adjustment to illness, consecutively.

The findings of our study showed that there was a highly significant improvement in healthcare orientation and vocational environment subdomains of psychological adjustment to illness in pre and post program implementation. In pre-program implementation less than half of the patients had good level of healthcare orientation and vocational environment that rose to more than three quarters of them having good level of healthcare orientation and more than two-thirds having good vocational environment level in post program evaluation. This displays the effectiveness of our program that addressed health education about disease and its management as well as, therapeutic communication. These findings correspond with the findings of Alnahas et al.^4^ and Al Salmi et al.^16^, whose findings revealed that the majority of hemodialysis patients had high healthcare orientation and had good vocational environment level, successively.

Concerning the domestic environment and extended family relationships subdomains of psychological adjustment to illness, our results revealed a highly significant improvement before and after the program implementation. Prior to the program, fewer than half of the patients reported high levels in both areas, while three-quarters of patients demonstrated improvement in the post-program evaluation. This suggests the effectiveness of our psychoeducational program, particularly the communication techniques that promote empathetic interactions with family members.

These results, however, differ from those of Zorba et al.^17^, who found poor extended family relationships among hemodialysis patients. Several factors may account for this discrepancy, such as differences in demographic characteristics. The patients in our study had been undergoing hemodialysis for less than a year, whereas those in Zorba et al.’s study had been receiving dialysis for a longer period. It is possible that the longer duration of treatment could have led to more entrenched family dynamics or emotional fatigue, potentially hindering relationship improvements. Additionally, cultural or regional differences in family structures and expectations may also play a role in the varying results.

In addition, our study findings revealed that there is a highly significant improvement in social environment and psychological distress subdomains of psychological adjustment to illness, in pre and post program implementation. In pre-program implementation, the majority of studied patients had poor social environment and psychological distress subdomain, which improved to be good level of both in post-program evaluation. The observed improvements suggest that targeted psychoeducational interventions can play a crucial role in alleviating psychological distress and enhancing the social environment for hemodialysis patients. It underscores the need for continued focus on personalized coping strategies to address the complex emotional and social challenges faced by these individuals. These findings corresponds with the findings of Sein et al.^18^ whose findings showed that the majority of hemodialysis patients had multiple sources of psychological distress. However, our findings contradict the findings of Tayea, et al.^19^ whom findings showed that the majority of the patients had good social environment.

Finally, the findings of our study revealed a highly significant correlation between self-efficacy and psychological adjustment to illness among hemodialysis patients. This is consistent with the findings of Mohamed et al.^20^, who found a highly significant positive correlation between self-efficacy and adherence to the therapeutic regimen among the studied patients.

Based on these findings, it is recommended that psychoeducational nursing programs to be integrated into the routine care of newly diagnosed hemodialysis patients to enhance their self-efficacy and psychological adjustment. The data highlight the importance of early psychological support interventions, particularly at the start of dialysis treatment when patients are most vulnerable to emotional distress. Future programs should also focus on individualized coping strategies, emotional regulation skills, and problem-solving abilities. Additionally, these interventions can be adapted for use in outpatient clinics, dialysis centers, and home-based care settings to promote long-term patient well-being and treatment adherence.

## Limitation of the study

Despite the significant findings, this study has some limitations. First, the study was conducted in a single setting with a relatively small sample size, which may limit the generalizability of the results to broader hemodialysis populations. Second, the short duration of follow-up after the psychoeducational program implementation did not allow assessment of the long-term sustainability of the improvements in self-efficacy and psychological adjustment. Third, self-reported questionnaires were used to measure outcomes, which may be subject to social desirability bias. Future studies with larger, multi-settings samples and longer follow-up periods are recommended to validate and expand upon these findings.

## Methods

### Research design

This study employed a quasi-experimental, single-group pre/post-test design to evaluate the effectiveness of the psychoeducational nursing program on self-efficacy and psychological adjustment among new hemodialysis patients.

### Setting and participants

This study was conducted in the hemodialysis units of the National Institute of Urology and Nephrology (NIUN), affiliated to the General Organization of Teaching Hospitals and Institutions, Cairo, Egypt. A purposive sample of [53] participants was selected based on the following inclusion criteria: patients undergoing hemodialysis for less than one year, aged 18–60 years, and free from diagnosed psychiatric disorders. Psychiatric conditions were ruled out based on patients’ medical records and clinical history.

### Fieldwork (intervention)

The psychoeducational nursing program was implemented by the researcher who is a professional nurse with expertise in hemodialysis management and is a PhD candidate in psychiatric and mental health nursing, over six months (September 2023–March 2024). A total of 53 participants were divided into six subgroups, with the intervention delivered separately to accommodate the setting capacity and patients’ schedules. Each subgroup attended 12 sessions over six weeks, with sessions held three times per week after dialysis. Each session lasted between 45 and 60 min. The program, designed by the researcher based on recent literature, was delivered in simple Arabic to ensure accessibility. It consists of two parts:

#### Part I: theoretical part

It was covered under four sessions that focused on providing basic information about hemodialysis and psychological reactions to illness, general guidelines for managing health and psychological issues, adaptation strategies, and enhancing self-efficacy among hemodialysis patients.

#### Part II: practical part

It was covered in eight that focused on practical guidelines for addressing psychological problems, techniques for managing negative emotions, engaging in therapeutic communication, enhancing self-efficacy, and managing stress and adaptation to hemodialysis.

The program content was delivered over 12 sessions, with each session lasting 45–60 min. The researcher employed various teaching methods and media, including open discussions, brainstorming, demonstrations, role-playing, videos, and handouts. At the beginning of each session, the researcher greeted the patients, provided feedback on the previous session, and explained the objectives of the new topic to ensure the patients understood the program content. At the end of each session, the patients’ questions were addressed to correct any misunderstandings. By the end of the program, the hemodialysis patients had acquired the necessary knowledge and skills to improve their adaptation and self-efficacy. This was achieved through:

- **Session (1):** Orientation session: In this session, the researcher established a rapport with the patient, explained the program agenda and meeting time to the patients, and pre-program assessment was done.
- **Sessions (2 & 3)** included an overview of hemodialysis, various psychological reactions to illness, and the stressors faced by new hemodialysis patients. This content was delivered using handouts, group discussions, and brief lectures.
- **Session (4)** focused on the definition and significance of coping, along with various types of coping strategies. The material was presented through handouts, group discussions, and brief lectures.
- **Session (5)** covered techniques for addressing negative emotions and maladaptive coping. The session employed group discussions, posters, small group activities, and role-playing to facilitate learning.
- **Session (6)** covered relaxation techniques through group discussions and demonstrations, utilizing small group activities, role-playing, and posters.
- **Session (7)** focused on the definition and importance as well as techniques for effective therapeutic communication. This was addressed through brief lectures, group discussions, handouts, and role-playing.
- **Session (8)** covered various techniques to enhance self-efficacy, through In brief lectures, group discussions, handouts, small group activities, and role-playing were utilized.
- **Session (9)** focused on the steps of problem-solving and case-demonstration. The content was delivered through group discussions, demonstrations, and handouts.
- **Session (10)** addressed puncture care and early signs of complications through demonstrations, group discussions, and handouts.
- **Session (11)** covered dietary and fluid restrictions as well as medication adherence instructions. This was facilitated through handouts and group discussions.
- **Session (12)** involved reviewing the content of the program and evaluating its effectiveness. This was conducted through group discussions and post-test questionnaire.

The researcher, a trained nursing professional with experience in patient education, developed the psychoeducational program based on recent literature and expert consultation. To ensure consistency, a structured session guide was used across all groups. Bias was minimized by maintaining a standardized delivery method, ensuring all participants received the same information and support. Additionally, patients were encouraged to actively participate and ask questions to enhance engagement while maintaining neutrality in facilitation.

### Tools of data collection/evaluation

Three tools were used in data collection and evaluation, which are:

- **Interviewing Socio-Demographic and clinical data Questionnaire:**
  It was designed by the researcher after reviewing related literature to assess socio demographic and clinical characteristics of hemodialysis patients, such as age, sex, date of diagnosis and number of sessions per week.
- **Psychological adjustment to illness scale (PAIS):**
  It is developed by Derogatis^21^, and was adapted by the researcher, to assess quality of a patient’s psychosocial adjustment to hemodialysis. It consists a 40-item, classified into 6 subdomains as follow: health care orientation, vocational environment, domestic environment, extended family relationships, social environment and psychological distress.
  **Scoring system:**
  Each items is rated on a 3-point Likert scale (1–3), score of even numbers are reversed, with higher rates indicating poorer psychological adjustment to illness. Scores (40–60) means Good psychological adjustment to illness, (61–90) indicates average psychological adjustment to illness & (90–120) indicates poor psychological adjustment to illness.
- **Self-efficacy for chronic kidney disease (CKD-SE) scale:**
  It is developed by Lin et al.^22^ and was adapted by the researcher to assess self-efficacy for managing chronic illness. It consists of 30 items classified into 5 sub-domains (autonomy, self-integration, problem solving, seeking social support& identifying and managing own emotions).
  **Scoring system:**
  Each item is rated on 3-point likert scale, with lower scores meaning poorer self-efficacy among hemodialysis patients. Scores (30–45) means low self-efficacy, (46– <68) indicates average self-efficacy and scores (68–90) indicates high self-efficacy.

### Statistical analysis

Data were analyzed using SPSS version 22.0 (SPSS Inc., Chicago, Illinois, USA). Quantitative variables were presented as mean ± standard deviation (SD), while qualitative variables were expressed as frequencies and percentages. Descriptive statistics were used for demographic and baseline characteristics. Chi-square and Pearson’s correlation coefficient tests were applied to examine associations between study variables.

### Validity and reliability

Validity and reliability of the study tools were assessed to ensure accuracy and consistency. Content validity was reviewed by a panel of five psychiatric and mental health nursing faculty experts, who evaluated the tools for clarity, relevance, comprehensiveness, simplicity, and applicability. Based on their feedback, minor modifications were made. For example, the ‘sexual environment’ domain was removed from the Psychological Adjustment to Illness Scale to align with cultural sensitivities. Language adjustments were also made to improve clarity and remove redundant phrasing.

Reliability was measured using Cronbach’s alpha, which assesses internal consistency. The Self-Efficacy for CKD Patients tool had a Cronbach’s alpha of 0.889, indicating excellent reliability. The Psychological Adjustment to Illness Scale had a Cronbach’s alpha of 0.722, while the overall total tool had a reliability coefficient of 0.789. All values exceeded the minimum required threshold of 0.60, confirming that the tools demonstrated good internal consistency. The content validity scores were 0.883 for the Self-Efficacy tool, 0.751 for the Psychological Adjustment to Illness Scale, and 0.801 for the total questionnaire, confirming the high validity and reliability of the instruments.

Before conducting inferential statistics, data normality was tested using [Shapiro–Wilk/Kolmogorov–Smirnov test] to ensure statistical assumptions were met. The results confirmed that data was normally distributed. Therefore, appropriate statistical tests, including Chi-square and Pearson’s correlation coefficient, were used based on data characteristics.

## Conclusion

Based on the implementation of the psychoeducational nursing program, there was a notable enhancement in self-efficacy and psychological adjustment to illness among new hemodialysis patients. Participants showed significant improvements in overall self-efficacy, particularly in the subdomains of autonomy, self-integration, problem-solving, and emotional management, with p-values less than 0.001. However, seeking social support did not show significant improvement. Psychological adjustment to illness also significantly improved, with marked enhancements in healthcare orientation, vocational environment, domestic environment, extended family relationships, social environment, and psychological distress subdomains (*p* < 0.001). There was a strong correlation between increased self-efficacy and better psychological adjustment, highlighting the program’s effectiveness.

## Data Availability

The associated data for this study is available upon reasonable request. Interested scholars can request the data by contacting the corresponding author at [helbasmah@gmail.com].

## References

[1] Özkan, İ & Taylan, S. Diet and fluid restriction experiences of patients on hemodialysis a meta-synthesis study. *Rev. Nefrol. Diálisis Traspl.* **42**, 22–40 (2022).

[2] Qalawa, S., Eltahry, S. & Aly, A. Self-efficacy among patients with hemodialysis during the COVID-19 pandemic. *J. Med. Life* **15**, 9. 10.53388/202209 (2023). 10.25122/jml-2021-0405 PMC9321481 35928350

[3] HassanpourDehkordi, A. et al. Empowerment and self-efficacy in patients with chronic disease a systematic review study. *J. Nephropharmacol.* **12**, e10596. 10.34172/npj.2023.10596 (2023).

[4] Alnahas, N., Shahin, E. & Bogdady, E. Health needs and self-efficacy for patients undergoing hemodialysis. *Port Said Sci. J. Nurs.* **10**, 2 (2023).

[5] Alkhaqani, A. L. Psychological impact of chronic kidney disease and hemodialysis narrative review. *Psychosom. Med. Res.* **2**, 1–5. 10.53388/202209 (2022).

[6] Stacherl, B. & Sauzet, O. Chronic disease onset and wellbeing development longitudinal analysis and the role of healthcare access. *Eur. J. Public Health* **34**, 29–34. 10.1093/eurpub/ckad070 (2023). 10.1093/eurpub/ckad167 PMC10843952 37802926

[7] Sreelatha, P. & Rajamani, M. Resilience and work and social adjustment in patients with end-stage renal disease undergoing hemodialysis. *Telangana J. Psychiatry* **9**, 49. 10.4103/tjp.tjp_53_22 (2023).

[8] Zhang, L., Zou, L. & Zhou, L. Effectiveness of psychoeducational interventions on psychological distress and health-related quality of life among patients with maintenance hemodialysis a systematic review and meta-analysis. *Ren. Fail.* **46**, 2331613. 10.1080/0886022X.2024.2331613 (2024). 38561244 10.1080/0886022X.2024.2331613 PMC10986446

[9] Farag, Y. & El-Sayed, E. Global dialysis perspective Egypt. *Kidney360* **3**, 1263–1268. 10.34067/KID.0007482021 (2022). 35919518 10.34067/KID.0007482021 PMC9337895

[10] Nuñez, B. Sociodemographic and clinical profile adherence to treatment and perceived social support among hemodialysis patients. *MJN* **15**, 4. 10.31674/mjn.2024.v15i04.015 (2024).

[11] Lee, M. et al. Effectiveness of a self-management program in enhancing quality of life self-care and self-efficacy in patients with hemodialysis a quasi-experimental design. *Semin. Dial.* **00**, 1–8. 10.1111/sdi.12957 (2021). 10.1111/sdi.12957 33533048

[12] Almutary, H. & Tayyib, N. Evaluating self-efficacy among patients undergoing dialysis therapy. *Nurs. Rep.* **11**, 195–201. 10.3390/nursrep11010020 (2021). 34968324 10.3390/nursrep11010019 PMC8608120

[13] Hafezieh, A., Denghan, M. & Iranmanesh, S. Self-management self-efficacy and knowledge among patients under haemodialysis a case in Iran. *J. Res. Nurs.* **25**, 128–138. 10.1177/1744987119874824 (2020). 34394617 10.1177/1744987120904770 PMC7932209

[14] Barello, S. et al. The effect of psychosocial interventions on depression anxiety and quality of life in hemodialysis patients a systematic review and a meta-analysis. *Int. Urol. Nephrol.* **55**, 897–912. 10.1007/s11255-022-02978-7 (2022). 36180655 10.1007/s11255-022-03374-3 PMC10030538

[15] Kapadi, R. et al. An exploration of successful psychosocial adjustment to long-term in-centre haemodialysis. *Psychol. Health*10.1080/08870446.2023.2231007 (2023). 37415316 10.1080/08870446.2023.2231007

[16] Al Salmi, I. et al. Kidney disease-specific quality of life among patients on hemodialysis. *Int. J. Nephrol.*10.1155/2021/8876559 (2021). 33880190 10.1155/2021/8876559 PMC8049780

[17] Zorba, E. et al. Investigation of social constraints psychosocial adjustment and optimism among dialysis patients. *Clin. Pract.* **14**, 1430–1439. 10.3390/clinpract140301430 (2024). 39051309 10.3390/clinpract14040115 PMC11270319

[18] Sein, K. et al. Emotional distress and adjustment in patients with end-stage kidney disease a qualitative exploration of patient experience in four hospital trusts in the West Midlands UK. *PLoS ONE* **15**, e0241629. 10.1371/journal.pone.0241629 (2020). 33152018 10.1371/journal.pone.0241629 PMC7644018

[19] Tayea, K., Hussein, M., Khalil, B. & El Wasif, S. Effect of hemodialysis long life program on the quality of life of patients with end-stage renal disease. *Eur. J. Healthc.* **13**, 2 (2022).

[20] Mohamed, H., Abdalla, K. & Khalifa, A. Relation between self-efficacy and adherence to therapeutic regimen among patients with post COVID-19 syndrome. *Eur. J. Healthc.* **15**, 1 (2024).

[21] Derogatis, L. R. The psychosocial adjustment to illness scale (PAIS). *J. Psychosom. Res.* **30**, 77–91. 10.1016/0022-3999(86)90069-3 (1986). 3701670 10.1016/0022-3999(86)90069-3

[22] Lin, C. et al. The chronic kidney disease self-efficacy (CKD-SE) instrument development and psychometric evaluation. *Nephrol. Dial. Transplant.* **27**, 3828–3834. 10.1093/ndt/gfr788 (2012). 22344776 10.1093/ndt/gfr788 PMC3808692

